# Outstanding Reviewers for *RSC Advances* in 2025

**DOI:** 10.1039/d6ra90053d

**Published:** 2026-07-06

## Abstract

We would like to take this opportunity to thank all of *RSC Advances*' reviewers for helping to preserve quality and integrity in chemistry literature. We would also like to highlight the Outstanding Reviewers for *RSC Advances* in 2025.

## Abstract 2

We would like to take this opportunity to highlight the Outstanding Reviewers for *RSC Advances* in 2025, as selected by the editorial team for their significant contribution to the journal.

## Graphical abstract

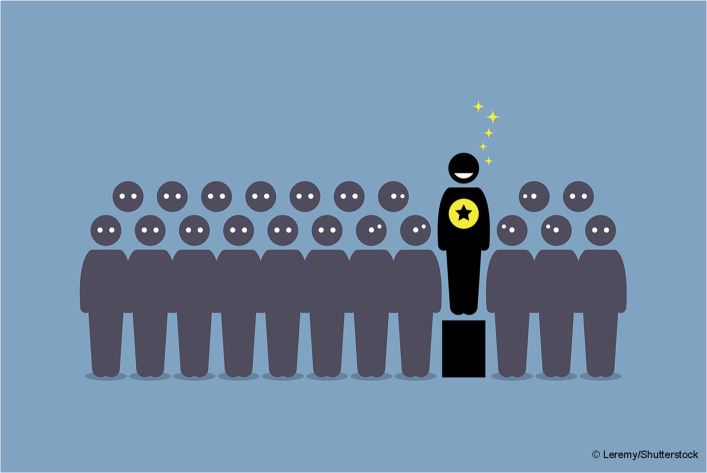

Each one of our outstanding peer reviewers has been carefully selected by our editorial team, and the list includes active researchers who have made significant contributions to peer review and have gone above and beyond in their actions. In recognition of the varied contributions of our reviewer community, our Outstanding Reviewers from 2025 have been chosen based on several different measures, including the number, timeliness, and quality of the reports completed during 2025. We also highlight reviewers who have provided exceptionally thorough and detailed reports, as well as reviewers who have made additional efforts to aid authors in improving their manuscripts.

We would also like to direct a special thanks to the members of the *RSC Advances* Reviewer Panel for their hard work and dedication, and the valuable contribution they have made to the journal. The Reviewer Panel is a key part of our commitment to deliver rigorous and fair peer review and ensures that manuscripts are handled by experts throughout the peer review process. We are proud to work with these individuals and recognise their crucial role for the journal.

*RSC Advances* strives to represent the diversity of the chemistry community, and we would like our reviewer community to reflect that. We are always looking to improve the diversity of our reviewer community in all its forms, and welcome applications for new reviewers through the Royal Society of Chemistry’s Become a Reviewer page (https://www.rsc.org/publishing/journals/review-for-us).

“*Reviewing is the first step of the scientific conversation that flows from publication in RSC Advances. We appreciate the insight and hard work of our reviewers in initiating these conversations that are the real strength of a society publisher like the RSC*” – Russell J. Cox, Editor-in-Chief, *RSC Advances*.

“*Peer review is central to the scientific integrity of RSC Advances, and we greatly appreciate all the effort that goes into the peer review process by our reviewers, ensuring rigour and quality across our publications, strengthening them through expert evaluation*” – Karen Faulds, Editor-in-Chief, *RSC Advances*.

***RSC Advances* 2025 Outstanding Reviewers:**

Dr Takumi Abe

Okayama University

ORCID: 0000-0003-1729-1097

Dr Prashanth Adarakatti

SVM Arts Science and Commerce College

ORCID: 0000-0002-9049-4862

Dr Akbar Ali

Government College University Faisalabad

ORCID: 0000-0002-2914-0934

Dr Raghavender Boda

Nanosyn Inc

ORCID: 0009-0001-6857-4671

Professor Søren Christensen

University of Copenhagen

ORCID: 0000-0002-5773-6874

Dr Poushali Das

McMaster University

ORCID: 0000-0002-0353-4396

Dr Lihua Gan

Tongji University

ORCID: 0000-0002-3652-8822

Dr Miodrag Lukic

University of Belgrade

ORCID: 0000-0002-4981-9776

Dr Hoang Van Ngoc

Ton Duc Thang University

ORCID: 0009-0006-1026-129X

Dr Victor Magno Paiva

Federal University of Rio de Janeiro

ORCID: 0000-0002-3390-2970

Dr Sharmiladevi Ramamoorthy

University of Verona

ORCID: 0000-0001-9784-0695

Dr Shubham Roy

Harbin Institute of Technology

ORCID: 0000-0001-5245-3229

Dr Siddiq Pasha Shaik

University of Nebraska-Lincoln

ORCID: 0009-0001-3907-9575

Dr Manisha Skaria

Yale University

ORCID: 0000-0002-6729-714X

Dr Yurij Stetsyshyn

Lvivska Polytechnika National University

ORCID: 0000-0002-6498-2619

Professor Shuning Xiao

University of Shanghai for Science and Technology

ORCID: 0000-0002-1312-9386

Dr Muzakkir Zainol

Universiti Teknologi MARA

ORCID: 0000-0003-0893-541X

***RSC Advances* Reviewer Panel 2025 Outstanding Reviewers:**

Dr Elias Antoine Akoury

Lebanese American University

ORCID: 0000-0001-5202-8935

Dr Haruyasu Asahara

Osaka University

ORCID: 0000-0002-1808-7373

Dr Debmalya Bhunia

Indian Institute of Chemical Biology CSIR

ORCID: 0000-0001-8649-0466

Dr Alien Blanco Fores

Tecnologico de Estudios Superiores de Tianguistenco

ORCID: 0000-0003-4035-0550

Professor Wanhao Cai

Hefei University of Technology

ORCID: 0000-0002-3466-8530

Dr Jin Choi

Odyssey Therapeutics Inc

ORCID: 0000-0001-5759-8419

Dr Shishir Chourey

Bristol-Myers Squibb Co R&D Lawrenceville

ORCID: 0000-0002-2629-9632

Dr Gui-Ping Dai

North Carolina Central University

ORCID: 0000-0001-9208-9994

Professor Feng Li

The University of Sydney

ORCID: 0000-0003-4448-074X

Dr Maria Vesna Nikolic

University of Belgrade Institute for Multidisciplinary Research

ORCID: 0000-0001-5035-0170

Dr Alexander Pokutsa

Department of Phisical Chemistry of Fuel

ORCID: 0000-0001-5564-1997

Dr Prathap Reddy Muktapuram

University of Cincinnati

ORCID: 0000-0002-9827-491X

Dr Uma B Reddy

Vijayanagara Sri Krishnadevaraya University

ORCID: 0000-0002-6745-2111

Dr Anindita Sarkar

University of Michigan

ORCID: 0000-0002-7947-860X

Dr Mohammad R. Thalji

Korea Institute of Energy Technology

ORCID: 0000-0002-0946-6136

Dr Rafael Vargas-Bernal

Polytechnic University of Guanajuato

ORCID: 0000-0003-4865-4575

Dr Zaka Ullah

University of Education

ORCID: 0000-0002-2321-8323

Dr Gang Yuan

Anhui University of Science and Technology

ORCID: 0000-0002-8098-8228

Dr Peng Zuo

Purdue University

ORCID: 0000-0003-1524-3200

We would also like to thank the *RSC Advances* Editorial Board and Associate Editors and the chemistry community for their continued support of the journal, as authors, reviewers and readers.

We continue to work on improving the diversity of our reviewer pool to reflect the diversity of the communities that we serve.

Russell J. Cox, Editor-in-Chief, Leibniz Universität Hannover, Germany

Karen Faulds, Editor-in-Chief, University of Strathclyde, UK

Laura Fisher, Executive Editor, Royal Society of Chemistry, UK

